# Association between hemoglobin-to-creatinine ratio and all-cause mortality in patients with coronary artery disease

**DOI:** 10.1186/s43044-025-00707-2

**Published:** 2025-12-05

**Authors:** Bo Kang, Chun Zhang, Jie Li, Liang Chen

**Affiliations:** 1 Fuwai Hospital, National Center of Cardiovascular Diseases, Chinese Academy of Medical Sciences & Peking Union Medical College, Beijing, China; 2 Kaifeng155 Hospital, China RongTong Medical Healthcare Group Co.Ltd, Kaifeng, China; 3 Ruijin-Hainan Hospital Shanghai Jiao Tong University School of Medicine, Hainan Boao Research Hospital, Qionghai, China; 4 The Second Affiliated Hospital of Dalian Medical University, Dalian, China

**Keywords:** Hemoglobin-to-creatinine ratio, Coronary artery disease, All-cause mortality, NHANES, Prognostic factors

## Abstract

**Background:**

Although the hemoglobin-to-creatinine ratio (HCR) has been recognized as a predictor for various diseases, its prognostic value in patients with coronary artery disease (CAD) remains unclear. This study aimed to examine the association between HCR and all-cause mortality in general CAD patients using data from the National Health and Nutrition Examination Survey (NHANES) database.

**Methods:**

A total of 3701 adult patients with CAD, which was identified through self-report in questionnaires, were included and followed for a mean of 79 months. The association between HCR and all-cause mortality was evaluated using smooth curve fitting, threshold effect analysis, and a competing risk regression model.

**Results:**

A nonlinear association was observed between HCR and all-cause mortality among CAD patients, characterized by an inflection point at 18.32. Below this threshold, each unit decrease in HCR was associated with a 6% reduction in all-cause mortality (HR = 0.94, 95%CI: 0.92–0.97, *p* < 0.001). Above the inflection point, each unit increase in HCR corresponded to a 7% increase in mortality risk (HR =1.07, 95%CI: 1.02–1.12, *p* = 0.005). The competing risk model revealed a similar association between HCR and cardiac mortality.

**Conclusions:**

Utilizing data from the nationally representative NHANES database, this study identified a nonlinear correlation between HCR and all-cause mortality in a general population of patients with CAD. Specifically, mortality risk initially decreased and subsequently increased with rising HCR levels, highlighting the broad relevance of these findings to community-based CAD management.

**Supplementary Information:**

The online version contains supplementary material available at 10.1186/s43044-025-00707-2.

## Introduction

As a major contributor to major adverse cardiovascular events, coronary artery disease (CAD) remains a major global cause of mortality and disability [1, 2]. In 2022 alone, CAD accounted for over 9.2 million deaths, imposing substantial healthcare and economic burdens worldwide [3–5]. Global CAD morbidity and mortality are anticipated to increase over the coming decades [1]. This growing burden highlights the urgent need to identify effective prognostic markers for the disease.

The hemoglobin-to-creatinine ratio (HCR), defined as hemoglobin divided by creatinine, is an affordable and readily accessible biomarker. Although HCR has been associated with adverse outcomes in many kinds of diseases [6–16], its prognostic value for all-cause mortality in CAD patients remains inadequately investigated. Both anemia and renal dysfunction are well-established independent risk factors for CAD, and their coexistence increases CAD risk [6–8, 12]. Similarly, patients undergoing percutaneous coronary intervention (PCI) with both anemia and renal impairment face a 3.9-fold higher mortality risk [7]. Although previous studies have demonstrated the prognostic value of the HCR in acute coronary syndromes and PCI populations [10, 11], to the best of our knowledge, its utility in broader CAD cohorts remains underexplored. Therefore, this study utilizes the NHANES database to assess the predictive value of HCR for all-cause mortality in individuals with CAD.

## Methods

### Study population

**Fig. 1:**
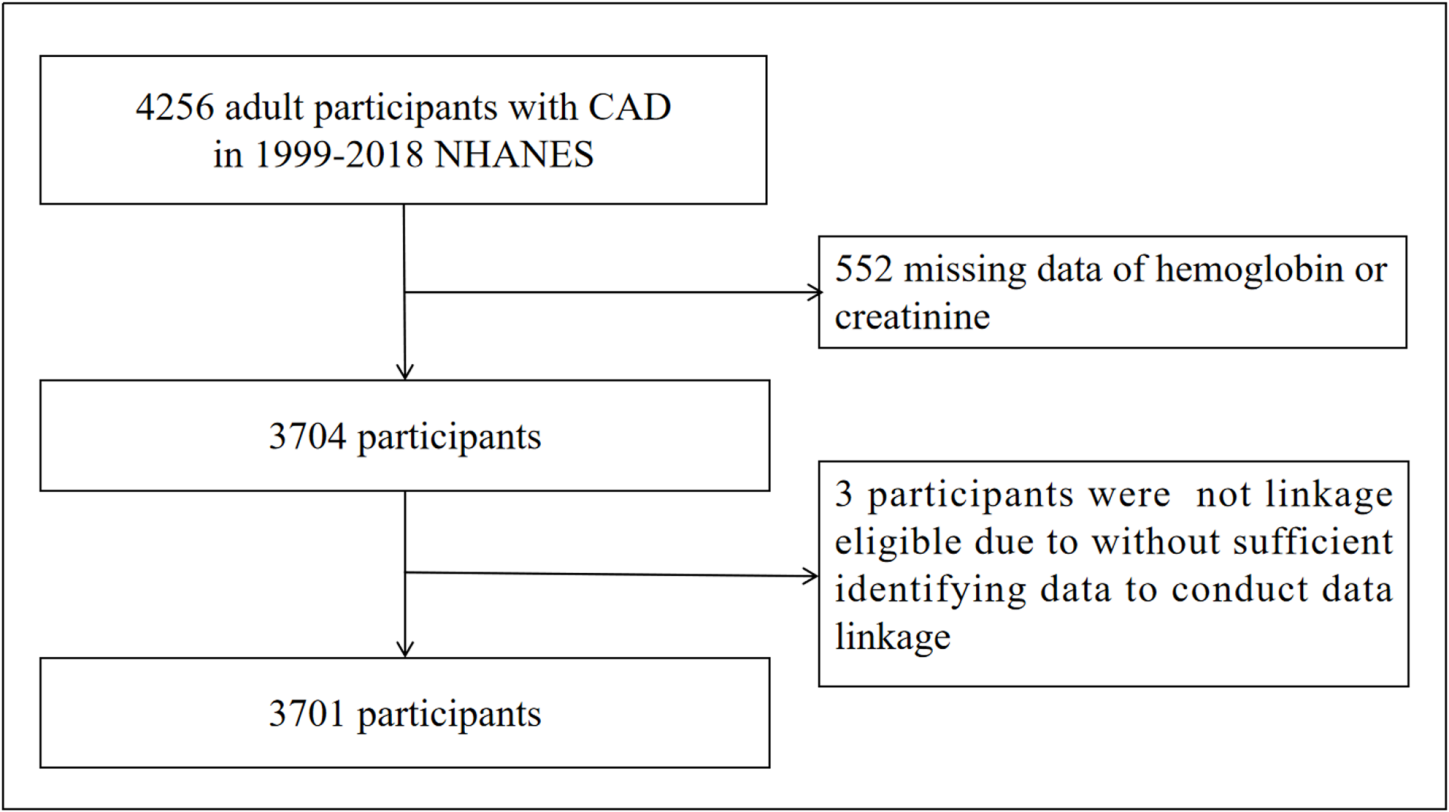
Flow diagram of the study selection process

This study utilized data from the National Health and Nutrition Examination Survey (NHANES). All participants provided written informed consent for the use of their data by researchers. From 1999 to 2018, 4256 adult patients with CAD from various races and regions were included in NHANES. Participants were defined as patients with CAD if they answered “yes” to the question of whether they had been told by doctors that they had CAD, angina pectoris, or myocardial infarction. After excluding participants with missing hemoglobin or creatinine data (*n* = 552) and those without linkage to the National Death Index (NDI) due to insufficient information (*n* = 3), a final cohort of 3701 participants was retained for analysis (Fig. 1).

### Outcome and covariates

#### Covariates

Baseline data were collected through computer-assisted interviews, questionnaires, and blood tests. Information demographic characteristics (age, sex, race, education level, and ratio of family income-to-poverty), risk factors (body mass index[BMI], smoking and alcohol use, history of hypertension, hypercholesterolemia, diabetes, heart failure, and stroke), and laboratory indices (hemoglobin, creatinine, uric acid, alanine, alanine aminotransferase[ALT], aspartate aminotransferase[AST], iron, and albumin). Patients with missing data for BMI, education level, or the ratio of family income to poverty were coded as ‘Unknow’ in the analysis. Smoking was defined as consumption of at least 100 cigarettes per year, alcohol use was defined as consumption of at least 12 alcoholic drinks within past 12 months [17]. Patients with a history of hypertension, hypercholesterolemia, diabetes, heart failure, or stroke were defined as answering “yes” to the question of ever having been told by doctors that they had the disease. Laboratory indices were derived from serum tests. The HCR was calculated by dividing hemoglobin by creatinine, with both components measured in standard units, and HCR was presented as a continuous variable. No apparent bias was observed in the data.

#### Outcome and follow-up data

The National Center for Health Statistics, operated by the U.S. government, links survey data with death certificate records from the NDI to examine associations between various health factors and mortality. This study examined all-cause mortality and cardiovascular mortality as the primary endpoints. The mortality outcomes used in this study were obtained from the official website (https://wwwn.cdc.gov/nchs/nhanes/Default.aspx).

### Statistical analysis

Categorical variables were expressed as numbers (percentages), and continuous variables as medians (first and third quartiles). Group differences were assessed using the chi-square test for categorical variables and the rank-sum test for continuous variables. A smooth curve fitting model was applied to examine the relationship between HCR and all-cause mortality. An inflection point was identified and verified using threshold effect analysis and the log-likelihood ratio test. Competing risk regression was performed to assess associations with both cardiac and all-cause mortality. Prior to regression, multicollinearity was assessed via variance inflation factors (VIF), and all covariates had VIF values below 5 (Detailed results are provided in Supplemental Table 1). The weight of NHANES survey was considered in regression analysis. Three regression models were constructed to evaluate the association between HCR and outcomes: Model 1 was unadjusted, and Model 2 adjusted for age, sex, and race. In Model 3, in addition to the fixed demographic adjustments, further covariates were selected based on the principle of covariate screening. Specifically, a confounding factor was included in the regression model if the change in P-values exceeded 10% when comparing its value before and after its introduction into the model. Ultimately, the adjustment factors in Model 3 comprised sex, age, race, education level, ratio of family income-to-poverty, BMI, smoking, alcohol use, ALT, uric acid, albumin, iron, hypertension, hypercholesterolemia, diabetes, heart failure, and stroke.

The data was analyzed using the Empower and Stata software. A *p*-value < 0.05 was considered as statistically significant.

## Results

### Baseline characteristics of participants

This study included 3701 adult patients with CAD, with a mean follow-up of 79 months. Among them, 2057 survived and 1644 died. Detailed baseline characteristics are presented in Table 1.

**Table 1:** Baseline characteristics of 3701 patients

Number	All	Q1 (1–12)	Q2 (12–18)	Q3 (18–21)	Q4 (21–75)	*P*
	3701	1084	1809	437	371	
Outcome						< 0.001
Survival	2057 (55.6%)	456 (42.1%)	1065 (58.9%)	304 (69.6%)	232 (62.5%)	
Death	1644 (44.4%)	628 (57.9%)	744 (41.1%)	133 (30.4%)	139 (37.5%)	
Time, months	79.0 (40.0-132.0)	61.0 (29.8–103.0)	83.0 (44.0-135.0)	109.0 (55.0-149.0)	103.0 (53.5-156.5)	< 0.001
Age, years	69.0 (60.0–78.0)	75.0 (67.0–80.0)	68.0 (60.0–77.0)	63.0 (53.0–72.0)	60.0 (51.0–70.0)	< 0.001
Sex						< 0.001
Male	2275 (61.5)	747 (68.9)	1197 (66.2)	212 (48.5)	119 (32.1)	
Female	1426 (38.5)	337 (31.1)	612 (33.8)	225 (51.5)	252 (67.9)	
Race						< 0.001
Mexican American	439 (11.9)	83 (7.7)	190 (10.5)	71 (16.2)	95 (25.6)	
Other Hispanic	242 (6.5)	55 (5.1)	108 (6.0)	42 (9.6)	37 (10.0)	
Non-Hispanic White	2192 (59.2)	637 (58.8)	1131 (62.5)	237 (54.2)	187 (50.4)	
Non-Hispanic Black	608 (16.4)	257 (23.7)	273 (15.1)	48 (11.0)	30 (8.1)	
Other Race	220 (5.9)	52 (4.8)	107 (5.9)	39 (8.9)	22 (5.9)	
Education level						< 0.001
< High school	1303 (35.2)	380 (35.1)	588 (32.5)	157 (35.9)	178 (48.0)	
High school graduate or general equivalency diploma	896 (24.2)	261 (24.1)	446 (24.7)	106 (24.3)	83 (22.4)	
>High school	1494 (40.4)	437 (40.3)	773 (42.7)	174 (39.8)	110 (29.6)	
Unknow	8 (0.2)	6 (0.6)	2 (0.1)	0 (0.0)	0 (0.0)	
Ratio of family income to poverty						< 0.001
≤ 1	754 (20.4)	190 (17.5)	339 (18.7)	107 (24.5)	118 (31.8)	
1–3	1686 (45.6)	536 (49.4)	824 (45.6)	186 (42.6)	140 (37.7)	
>3	954 (25.8)	266 (24.5)	508 (28.1)	105 (24.0)	75 (20.2)	
Unknow	307 (8.3)	92 (8.5)	138 (7.6)	39 (8.9)	38 (10.2)	
BMI						< 0.001
<30 kg/m2	1843 (49.8)	519 (47.9)	905 (50.0)	219 (50.1)	200 (53.9)	
≧ 30 kg/m2	1409 (38.1)	394 (36.3)	685 (37.9)	178 (40.7)	152 (41.0)	
Unknow	449 (12.1)	171 (15.8)	219 (12.1)	40 (9.2)	19 (5.1)	
Smoking						0.08
No	1393 (37.6)	436 (40.2)	644 (35.6)	171 (39.1)	142 (38.3)	
Yes	2308 (62.4)	648 (59.8)	1165 (64.4)	266 (60.9)	229 (61.7)	
Alcohol use						< 0.001
No	1487 (40.2)	480 (44.3)	658 (36.4)	177 (40.5)	172 (46.4)	
Yes	2214 (59.8)	604 (55.7)	1151 (63.6)	260 (59.5)	199 (53.6)	
Hypertension						< 0.001
No	1052 (28.4)	203 (18.7)	561 (31.0)	151 (34.6)	137 (36.9)	
Yes	2649 (71.6)	881 (81.3)	1248 (69.0)	286 (65.4)	234 (63.1)	
Hypercholesterolemia						0.012
No	1411 (38.1)	390 (36.0)	675 (37.3)	181 (41.4)	165 (44.5)	
Yes	2290 (61.9)	694 (64.0)	1134 (62.7)	256 (58.6)	206 (55.5)	
Diabetes						< 0.001
No	2518 (68.0)	635 (58.6)	1299 (71.8)	328 (75.1)	256 (69.0)	
Yes	1183 (32.0)	449 (41.4)	510 (28.2)	109 (24.9)	115 (31.0)	
Heart Failure						< 0.001
No	2701 (73.0)	673 (62.1)	1388 (76.7)	347 (79.4)	293 (79.0)	
Yes	1000 (27.0)	411 (37.9)	421 (23.3)	90 (20.6)	78 (21.0)	
Stroke						< 0.001
No	3086 (83.4)	850 (78.4)	1536 (84.9)	384 (87.9)	316 (85.2)	
Yes	615 (16.6)	234 (21.6)	273 (15.1)	53 (12.1)	55 (14.8)	
Uric Acid, umol/L	350.9 (291.5-416.4)	404.5 (339.0-475.8)	345.0 (291.5-398.5)	309.3 (261.7-362.8)	291.5 (243.9–339.0)	< 0.001
ALT, U/L	23.0 (17.0–40.0)	21.0 (15.0–37.0)	24.0 (17.0–40.0)	24.0 (18.0–43.0)	24.0 (18.0-41.8)	< 0.001
AST, U/L	23.0 (19.0–28.0)	22.0 (19.0–27.0)	23.0 (20.0–28.0)	23.0 (19.8–28.0)	23.0 (19.0–28.0)	0.018
Hemoglobin, g/dL	14.0 (12.9–15.0)	13.0 (12.0-14.1)	14.3 (13.3–15.2)	14.5 (13.6–15.4)	14.5 (13.6–15.4)	< 0.001
Creatinine, mg/dL	1.0 (0.8–1.2)	1.4 (1.2–1.7)	1.0 (0.9–1.1)	0.8 (0.7–0.8)	0.6 (0.6–0.7)	< 0.001
HCR	14.4 (11.3–17.6)	9.6 (7.7–10.8)	14.8 (13.5–16.3)	19.1 (18.5–19.9)	23.2 (22.0-25.3)	< 0.001

### The relationship of HCR and mortality

**Fig. 2:**
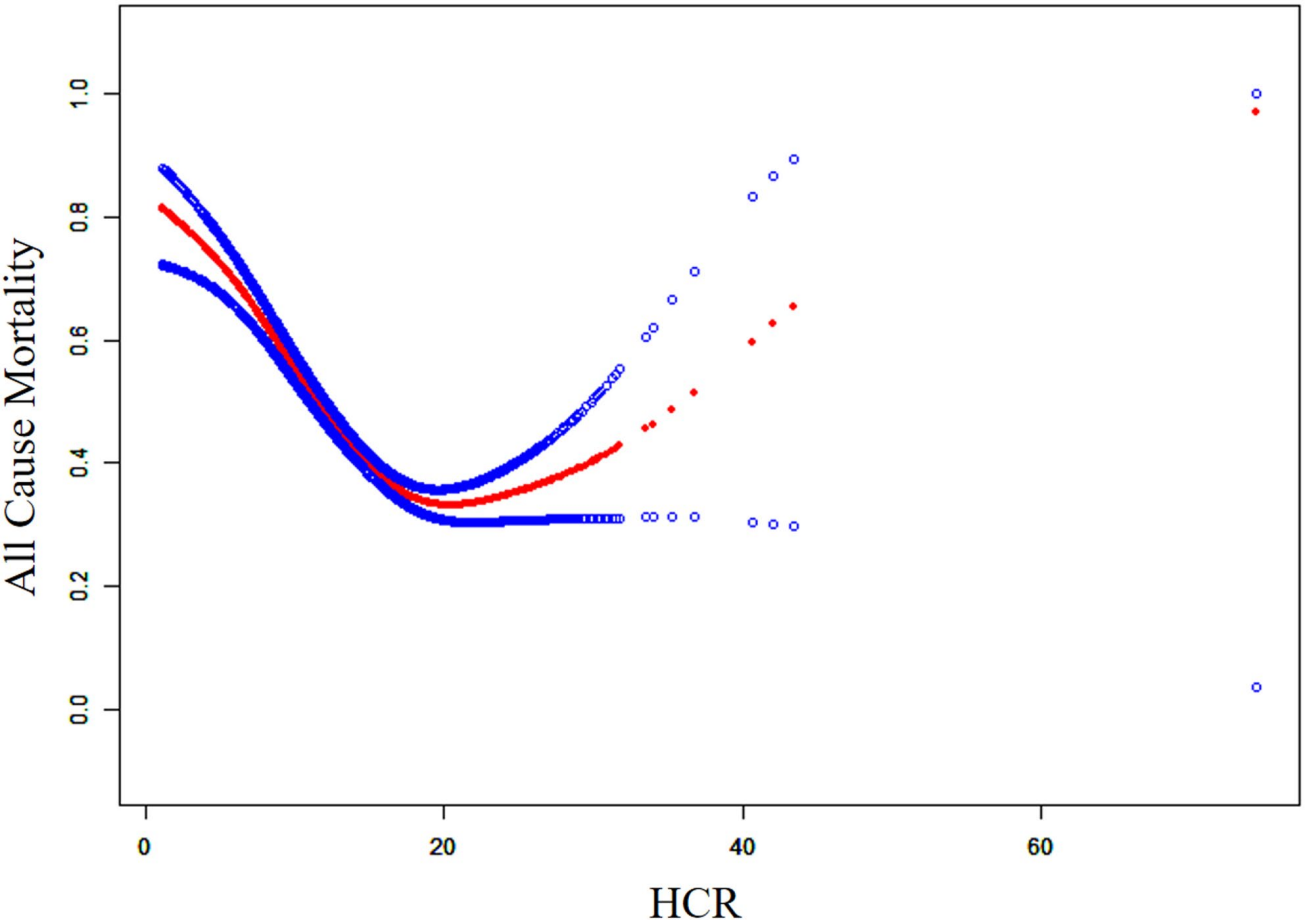
Smooth curve fitting of HCR and all-cause mortality in patients with CAD

As shown in Fig. 2, a distinct inflection point in the smooth curve indicates a nonlinear relationship between HCR and all-cause mortality in CAD patients. Consequently, threshold effect analysis and a log-likelihood ratio test were conducted. The results suggest that the inflection point is at 18.32. In the fully adjusted Model 3, each unit decrease in HCR below 18.32 was associated with a 6% reduction in mortality risk (HR = 0.94, 95%CI: 0.92–0.97, *p* < 0.001). Conversely, each unit increase in HCR above 18.32 corresponded to a 7% increase in risk (HR = 1.07, 95%CI: 1.02–1.12, *p* = 0.005). Detailed results are provided in Table 2. Subgroup analysis suggests that similar results are shown in different subgroups (Table 3).

Based on the inflection point, HCR was also categorized into quartiles for COX regression analysis. The results indicated that an HCR level between 18 and 21 was associated with the lowest mortality risk (HR = 0.70, 95% CI: 0.55–0.88, *p =* 0.002). Further details are presented in Table 4.

**Table 2:** Threshold saturation effect analysis of HCR and all-cause mortality in patients with CAD (weighted)

	Model 1	Model 2	Model 3
	HR(95%CI)*P*	HR(95%CI)*P*	HR(95%CI)*P*
HCR < 18.32	0.87 (0.85, 0.88) < 0.001	0.91 (0.89, 0.93) < 0.001	0.94 (0.92, 0.97) < 0.001
HCR ≧ 18.32	1.03 (1.01, 1.06) 0.014	1.05 (1.02, 1.08) < 0.001	1.07 (1.02, 1.12) 0.005
*p* for Logarithmic likelihood ratio test	< 0.001	< 0.001	< 0.001

Model 1: no covariate was adjusted

Model 2: sex, age, and race were adjusted

Model 3: sex, age, race, education level, ratio of family income-to-poverty, BMI, smoking, alcohol use, ALT, uric acid, iron, albumin, hypertension, hypercholesterolemia, diabetes, heart failure, and stroke were adjusted

**Table 3:** Subgroup analysis

	HCR<18.32	HCR ≧ 18.32	*P* for Logarithmic likelihood ratio test
	HR(95%CI)*P*	HR(95%CI)*P*	
Sex			
Male	0.93 (0.89, 0.97)	1.04 (0.99, 1.10)	0.007
Femal	0.95 (0.93, 0.97)	1.06 (1.02, 1.09)	< 0.001
Age			
Age<65	0.96 (0.94, 0.98)	1.10 (1.04, 1.16)	< 0.001
Age ≥ 65	0.92 (0.89, 0.95)	1.04 (1.00, 1.08)	< 0.001
BMI			
<30 kg/m2	0.96 (0.93, 0.98)	1.06 (1.02, 1.10)	< 0.001
≧ 30 kg/m2	0.93 (0.90, 0.95)	1.04 (1.00, 1.09)	< 0.001
Hypertension			
No	0.98 (0.94, 1.02)	1.09 (1.03, 1.15)	0.007
Yes	0.94 (0.92, 0.96)	1.04 (1.00, 1.07)	< 0.001
Hypercholesterolemia			
No	0.95 (0.92, 0.98)	1.04 (0.98, 1.10)	0.013
Yes	0.95 (0.92, 0.97)	1.06 (1.02, 1.09)	< 0.001
Diabetes			
No	0.95 (0.93, 0.98)	1.08 (1.03, 1.12)	< 0.001
Yes	0.95 (0.92, 0.97)	1.03 (0.98, 1.08)	0.009
Heart Failure			
No	0.95 (0.93, 0.97)	1.05 (1.01, 1.09)	< 0.001
Yes	0.93 (0.90, 0.96)	1.05 (1.01, 1.10)	< 0.001
Stroke			
No	0.96 (0.94, 0.98)	1.05 (1.01, 1.08)	< 0.001
Yes	0.91 (0.87, 0.95)	1.07 (0.99, 1.16)	0.003

Adjusted for sex, age, race, education level, ratio of family income-to-poverty, BMI, smoking, alcohol use, ALT, uric acid, iron, albumin, hypertension, hypercholesterolemia, diabetes, heart failure, and stroke (except the stratification factor itself)

**Table 4:** Different levels of HCR and all-cause mortality in patients with CAD (weighted)

HCR Quartile	Model 1	Model 2	Model 3
	HR(95%CI)*P*	HR(95%CI)*P*	HR(95%CI)*P*
HCR <12	Ref.	Ref.	Ref.
HCR 12–18	0.43 (0.38, 0.49)<0.001	0.64 (0.56, 0.73)<0.001	0.79 (0.68, 0.91)<0.001
HCR 18–21	0.29 (0.24, 0.35)<0.001	0.54 (0.43, 0.67)<0.001	0.70 (0.55, 0.88) 0.002
HCR ≥ 21	0.38 (0.29, 0.49)<0.001	0.82 (0.64, 1.05) 0.113	1.15 (0.86, 1.52) 0.341

Model 1: no covariate was adjusted

Model 2: sex, age, and race were adjusted

Model 3: sex, age, race, education level, ratio of family income-to-poverty, BMI, smoking, alcohol use, ALT, uric acid, iron, albumin, hypertension, hypercholesterolemia, diabetes, heart failure, and stroke were adjusted

### Competing risk model analysis

In the competing risk model analysis, the results were consistent with those from the smooth curve fitting and threshold effect analysis. Specifically, when HCR was below 18.32, the risk of cardiac death gradually decreased with increasing HCR (SHR = 0.97, 95% CI: 0.94-1.00, *p* = 0.027). In contrast, above this threshold, the risk of cardiac death showed a gradual increase with higher HCR levels, although this association was not statistically significant (SHR = 1.02, 95% CI: 0.949–1.06, *p* = 0.242). Detailed results are provided in Table 5.

**Table 5:** Competing risk model analysis of HCR and cardiac and all-other-causes death

	HCR < 18.32	HCR ≧ 18.32
	SHR(95%CI)*p*	SHR(95%CI)*p*
Disease of heart	0.97(0.94,1.00)0.027	1.02(0.99,1.06)0.242
All other cause	0.95(0.92,0.97) < 0.001	1.01(0.99,1.04)0.349

Adjusted for sex, age, race, education level, ratio of family income-to-poverty, BMI, smoking, alcohol use, ALT, uric acid, hypertension, hypercholesterolemia, diabetes, heart failure, and stroke

## Discussion

Analysis of data from a nationally representative sample of general CAD patients in the NHANES cohort revealed a nonlinear association between HCR and all-cause mortality, characterized by an initial decline followed by an increase in mortality as HCR levels rose. Threshold analysis identified an inflection point at 18.32, and quartile-based regression further indicated that maintaining HCR within the range of 18–21 was associated with the lowest mortality. Consistent results were observed in competing risk models assessing cardiac mortality, reinforcing the generalizability of these findings to broader community-based CAD populations.

Our findings in present study align with a growing trend of novel blood-based biomarkers across diverse patient populations. For instance, in patients undergoing Transcatheter Aortic Valve Implantation, mortality was significantly associated with low hemoglobin and high creatinine levels [18]. Similarly, for chronic coronary syndrome [19], viral hepatitis [20] and even in the context of bariatric surgery [21], positive advances of blood-based indicators were shown in all of these populations. These emerging evidences signal a paradigm shift towards using diverse blood-based biomarkers to refine risk prediction in diseases and surgeries.

The HCR is an established prognostic marker in cardiovascular disease, reflecting the synergistic effect of hemoglobin and creatinine on CAD pathogenesis. The prognostic value of HCR is supported by two studies from the PRAISE registry [15, 16], which linked HCR to outcomes in acute coronary syndrome (ACS) and STEMI populations. A striking consistency was observed, with nearly identical optimal cut-points for mortality (13.68 and 13.79), reinforcing HCR’s robustness as a biomarker. In addition, Claudine et al. found a 2.7-fold increased CAD risk in patients with both elevated creatinine and anemia compared to elevated creatinine alone [6, 7], consistent with earlier findings by Ishigami et al. [8]. Further studies support HCR’s clinical utility: Demir et al. showed HCR independently predicts long-term mortality in ACS patients [10], while Numasawa et al. identified a nonlinear relationship in PCI patients, with risk escalating sharply once HCR falls below 10 [9]. Similar inverse correlations with mortality were confirmed in AMI patients post-PCI [11]. While previous studies have extensively validated HCR’s prognostic value in acute coronary syndromes (ACS, STEMI, AMI) and periprocedural settings (PCI), our study extends this evidence by specifically focusing on the long-term all-cause mortality in a broader cohort of patients with established CAD, thereby addressing a gap in the long-term prognostic assessment of stable CAD populations.

As a routine clinical measure, hemoglobin serves as a predictor of CAD and is associated with adverse cardiovascular outcomes, though some studies report conflicting results [22–25]. Reduced hemoglobin impairs myocardial perfusion, exacerbating ischemia in CAD [26, 27]. Meanwhile, anemia activates the sympathetic and renin–angiotensin–aldosterone systems, lowering vascular resistance while increasing cardiac output and heart rate; chronic anemia may further promote left ventricular remodeling and cardiomyocyte death [27–29]. Notably, confounding factors such as comorbidities in anemic patients may complicate these associations [26, 29]. Overall, current evidence highlights hemodynamic alterations and perfusion deficits as central mechanisms linking hemoglobin to CAD.

Creatinine is a key diagnostic marker for kidney disease [30, 31] and a established predictor of adverse cardiovascular outcomes. Elevated levels have been associated with one-year mortality in ACS patients [32] and with cardiac events in elderly CAD patients undergoing non-cardiac surgery [12]. Chronic kidney disease (CKD), even in early stages, significantly increases CAD risk and contributes to poor cardiovascular health through multiple pathways [33, 34]. In CKD patients, inflammatory states and oxidative stress are prevalent, with elevated levels of inflammatory factors, cytokines, and oxidation products adversely affecting cardiovascular outcomes [35, 36]. Dyslipidemia, a common complication of CKD, accelerates atherosclerosis [37]. Hyperhomocysteinemia, frequently observed in renal insufficiency, contributes to central and peripheral cardiovascular diseases [38, 39]. Additionally, CKD predisposes patients to thrombosis and bleeding due to a hypercoagulable state and impaired platelet function [40]. Moreover, CKD drives cardiac remodeling, leading to left ventricular hypertrophy, fibrosis, and capillary rarefaction, which further worsen cardiac dysfunction [36].

Anemia and renal dysfunction synergistically increase CAD risk through bidirectional pathways. Clinical studies show patients with both conditions face significantly higher risks - a 2.7-fold rise in CAD incidence [6] and fourfold increased post-PCI mortality [7]. This synergy arises through shared pathways including inflammation, endothelial dysfunction, and hypoxic injury [29, 41]. Renal dysfunction exacerbates anemia via erythropoietin suppression, inflammation-induced hyporesponsiveness to erythropoiesis-stimulating agents, and iron deficiency [42–44]. Conversely, anemia worsens renal outcomes through hypoxia-induced vasoconstriction, dysregulated angiogenesis, and fibrosis [45, 46]. This bidirectional interaction creates a vicious cycle [47, 48]. By integrating both pathways, the HCR provides superior prognostic value over either marker alone in CAD, especially in patients with concurrent anemia and renal impairment. Our additional analysis indicated that extreme levels of both hemoglobin and creatinine were associated with higher mortality (Supplemental Fig. 1, Supplemental Table 2). However, the receiver operating characteristic curve demonstrated a higher area under curve for the HCR than for either parameter alone (Supplemental Fig. 2), suggesting its superior predictive ability as a composite biomarker.

Smooth curve fitting revealed progressively increasing all-cause mortality beyond the HCR inflection point. Several potential mechanisms may contribute to this association, such as red blood cell-driven hyperviscosity promoting thromboembolism [49], hemoglobin-associated metabolic syndrome elevating mortality [50]. Besides, low creatinine may reflect lack of protein intake and lower muscle mass [51, 52], indicating a worse overall condition. Thus, both extremes of HCR correlate with heightened all-cause mortality in CAD. As a composite biomarker integrating hemoglobin-creatinine balance, HCR may reflect metabolic, nutritional, and organ damage status. These findings suggest a potential association between maintaining HCR within a specific range and improved outcomes in CAD, although further validation is needed before clinical recommendations can be established.

This study identifies HCR = 18.32 as a critical inflection point for all-cause mortality risk stratification in CAD patients, offering direct clinical utility: First, as an accessible composite biomarker integrating anemia and renal dysfunction signals, HCR outperforms isolated parameters in identifying high-risk subgroups warranting intensified intervention. Second, it defines distinct therapeutic windows. Below the threshold, each unit increase in HCR corresponds to a 6% reduction in mortality, supporting hemoglobin optimization or renal protection; above it, each unit increase elevates risk by 7%, necessitating caution against excessive hemoglobin elevation due to thromboembolic and metabolic hazards. Third, serial HCR monitoring is recommended to maintain levels within an optimal range, particularly in CAD patients with comorbid anemia or CKD, to mitigate cardiac remodeling and mortality. Finally, in resource-limited settings, HCR serves as a cost-effective triage tool to guide specialist referrals, thereby optimizing healthcare delivery while reducing economic burdens.

Overall, HCR is significantly associated with mortality in CAD, mediated through hypoxia-driven pathology, systemic inflammation, and cardiac remodeling. This association is strengthened by the synergistic interaction of anemia and renal dysfunction, highlighting HCR’s utility as a composite predictive marker. It’s accessibility and cost-effectiveness make it a highly promising biomarker for prognostic risk stratification.

## Limitations

This study has several limitations. First, the diagnosis of CAD was based solely on self-reported data, which is subject to recall bias and may lead to misclassification. Although we adjusted for a wide range of covariates, residual confounding cannot be ruled out due to unmeasured variables, including medication use (e.g., statins, erythropoiesis-stimulating agents, or renin-angiotensin system blockers), CAD severity, nutritional status, and inflammatory biomarkers. Additionally, the inherent biological variability of hemoglobin and creatinine levels underscores the need for longitudinal measurements to more accurately capture time-dependent variations in HCR and draw definitive conclusions. Finally, the generalizability of our findings requires further validation in broader, multi-regional CAD cohorts beyond the United States, while carefully considering relevant demographic differences. Future studies should validate this inflection point in independent, multi-ethnic cohorts with objectively confirmed CAD, and investigate whether targeting HCR within the optimal range improves clinical outcomes.

## Conclusion

This study found a non-linear relationship between HCR and all-cause mortality in CAD patients, where mortality initially decreased and then increased with rising HCR, exhibiting an inflection point at 18.32. Therefore, the accessibility and cost-effectiveness of HCR make it a highly promising biomarker for risk stratification. To advance these findings, future research must validate this inflection point in independent, multi-ethnic cohorts with objectively confirmed CAD and determine whether targeting HCR within the optimal range improves clinical outcomes.

## Supplementary Information

Below is the link to the electronic supplementary material.

Supplementary Material 1.

Supplementary Material 2.

## Data Availability

The data for this study was could be found in the Supplementary File, and the original data could be obtained from the official website of NHANES (https://wwwn.cdc.gov/nchs/nhanes/Default.aspx).

## Abbreviations

CAD: Coronary Artery Disease
HCR: Hemoglobin-to-Creatinine Ratio
PCI: Percutaneous Coronary Intervention
NHANES: National Health and Nutrition Examination Survey
NDI: National Death Index
BMI: Body Mass Index
ALT: Alanine aminotransferase
AST: Aspartate aminotransferase
VIF: Variance Inflation Factors
ACS: Acute Coronary Syndrome
CKD: Chronic Kidney Disease

## References

[1] Ralapanawa U, Sivakanesan R (2021) Epidemiology and the magnitude of coronary artery disease and acute coronary syndrome: A narrative review. J Epidemiol Global Health 11(2):169–177. 10.2991/jegh.k.201217.001 10.2991/jegh.k.201217.001 PMC8242111 33605111

[2] Chamberlain AM, Boyd CM, Manemann SM, Dunlay SM, Gerber Y, Killian JM et al (2020) Risk factors for heart failure in the community: differences by age and ejection fraction. Am J Med 133(6):e237–e248. 10.1016/j.amjmed.2019.10.030 31747542 10.1016/j.amjmed.2019.10.030 PMC7558500

[3] Bauersachs R, Zeymer U, Brière JB, Marre C, Bowrin K, Huelsebeck M (2019) Burden of coronary artery disease and peripheral artery disease: A literature review. Cardiovasc Ther 262019:8295054. 10.1155/2019/8295054 10.1155/2019/8295054 PMC7024142 32099582

[4] Mensah GA, Fuster V, Murray CJL, Roth GA (2023) Global burden of cardiovascular diseases and Risks, 1990–2022. J Am Coll Cardiol 82:2350–2473. 10.1016/j.jacc.2023.11.007 38092509 10.1016/j.jacc.2023.11.007 PMC7615984

[5] Odden MC, Coxson PG, Moran A, Lightwood JM, Goldman L, Bibbins-Domingo K (2011) The impact of the aging population on coronary heart disease in the united States. Am J Med 124(9):827–33e5. 10.1016/j.amjmed.2011.04.010 21722862 10.1016/j.amjmed.2011.04.010 PMC3159777

[6] Jurkovitz CT, Abramson JL, Vaccarino LV, Weintraub WS, McClellan WM (2003) Association of high serum creatinine and anemia increases the risk of coronary events: results from the prospective Community-Based atherosclerosis risk in communities (ARIC) study. J Am Soc Nephrol 14(11):2919–2925. 10.1097/01.Asn.0000092138.65211.71 14569102 10.1097/01.asn.0000092138.65211.71

[7] Pilgrim T, Rothenbühler M, Kalesan B, Pulver C, Stefanini GG, Zanchin T et al (2014) Additive effect of anemia and renal impairment on Long-Term outcome after percutaneous coronary intervention. PLoS ONE 9(12):e114846. 10.1371/journal.pone.0114846 25489846 10.1371/journal.pone.0114846 PMC4260949

[8] Ishigami J, Grams ME, Naik RP, Caughey MC, Loehr LR, Uchida S et al (2018) Hemoglobin, Albuminuria, and kidney function in cardiovascular risk: the ARIC (Atherosclerosis risk in Communities) study. J Am Heart Assoc 7(2):e007209. 10.1161/jaha.117.007209 29330257 10.1161/JAHA.117.007209 PMC5850152

[9] Numasawa Y, Inohara T, Ishii H, Yamaji K, Kohsaka S, Sawano M et al (2020) Association of the hemoglobin to serum creatinine ratio with In-Hospital adverse outcomes after percutaneous coronary intervention among Non-Dialysis patients: insights from a Japanese nationwide registry (J-PCI registry). J Clin Med 10(11):3612. 10.3390/jcm9113612 10.3390/jcm9113612 PMC7696709 33182592

[10] Demir M, Kahraman F, Sen T, Astarcioglu MA (2023) The relationship of the hemoglobin to serum creatinine ratio with long-term mortality in patients with acute coronary syndrome: A retrospective study. Med (Baltim) 102(41):e35636. 10.1097/md.0000000000035636 10.1097/MD.0000000000035636 PMC10578685 37832061

[11] Bao F, Yang C, Zhou G (2023) Predictive value of hemoglobin to serum creatinine ratio combined with serum uric acid for in-hospital mortality after emergency percutaneous coronary intervention in patients with acute myocardial infarction. Zhonghua Wei Zhong Bing Ji Jiu Yi Xue 35(9):951–957. 10.3760/cma.j.cn121430-20230418-00291 37803954 10.3760/cma.j.cn121430-20230418-00291

[12] Li X, Wang C, Jin Y (2024) Temporal trends and risk factors of perioperative cardiac events in patients over 80 years old with coronary artery disease undergoing noncardiac surgery: a high-volume single-center experience, 2014–2022. Postgrad Med J 100(1182):252–261. 10.1093/postmj/qgad141 38223919 10.1093/postmj/qgad141

[13] Çamci S, Kinik M, Ari S, Ari H, Melek M, Bozat T (2022) The predictive value of hemoglobin to creatinine ratio for contrast-induced nephropathy in percutaneous coronary interventions. Clin Chem Lab Med 60(9):1455–1462. 10.1515/cclm-2022-0247 35727209 10.1515/cclm-2022-0247

[14] Meagher M, Autorino R, Mehrazin R, Eun D, Margulis V, Uzzo R (2022) Elevated hemoglobin: creatinine ratio is a novel preoperative marker for worsened survival outcomes in upper tract urothelial carcinoma: analysis from the Robuust registry. Eur Urol 207:e264. 10.1097/JU.0000000000002547.06 10.23736/S2724-6051.25.05498-9 41410659

[15] Spadafora L, Mattia Galli MG, Marco Bernardi MB, Matteo Betti MB, Stefano Cacciatore SC, Fabrizio D’ascenzo FDA et al (2024) Hemoglobin/creatinine ratio as long- term prognostic marker in acute coronary syndromes: a praise registry study. Eur Heart J 10.1093/eurheartj/ehae666.1571

[16] Spadafora L, Cacciatore S, Galli M, Collet C, Betti M, Sarto G et al (2025) Hemoglobin-to-Creatinine ratio predicts One-Year adverse clinical outcomes in ST-Elevation myocardial infarction: retrospective and propensity score matched analysis. J Clin Med 17(8):2756. 10.3390/jcm14082756 10.3390/jcm14082756 PMC12027881 40283586

[17] Zeng YM, Chen YY, Li J, Chen L (2025) Nonlinear association between the serum uric acid-to-creatinine ratio and all cause mortality in patients with hypertension: a ten-year cohort study using the NHANES database. Sci Rep 14:31423. 10.1038/s41598-024-83034-x 10.1038/s41598-024-83034-x PMC11682339 39733075

[18] AziziKia H, Mousavi A, Shojaei S, Shaker F, Salabat D, Bahri RA et,al (2025) Predictive potential of pre-procedural cardiac and inflammatory biomarkers regarding mortality following transcatheter aortic valve implantation: A systematic review and meta-analysis. Heart Lung 69:229–240. 10.1016/j.hrtlng.2024.10.011 39509738 10.1016/j.hrtlng.2024.10.011

[19] Lohner V, Perna L, Schöttker B, Perneczky R, Brenner H, Mons U (2025) Associations of blood-based biomarkers of neurodegenerative diseases with mortality, cardio- and cerebrovascular events in persons with chronic coronary syndrome. Exp Gerontol 200:112684. 10.1016/j.exger.2025.112684 39824235 10.1016/j.exger.2025.112684

[20] Roudsari PP, Shojaei S, Firoozbakhsh P, Azarboo A, Mirmoosavi S, Moradi A et al (2025) Prognostic value of inflammatory markers including neutrophil to lymphocyte ratio, platelet to lymphocyte ratio, mean platelet volume, platelet distribution width, and red blood cell distribution width in viral hepatitis: a systematic review and meta-analysis. J Clin Virol Plus 10.1016/j.jcvp.2025.100229

[21] AziziKia H, Shojaei S, Mousavi A, Salabat D, Shaker F, Dolama RH et al (2024) Periprocedural changes of serum biomarkers in predicting complications following bariatric surgery for obesity: systematic review and Meta-analysis. Obes Surg 34(6):2198–2215. 10.1007/s11695-024-07234-0 38676847 10.1007/s11695-024-07234-0

[22] Doganer YC, Rohrer JE, Aydogan U, Bernard ME, Barcin C (2015) Haemoglobin levels correlates with the presence of coronary artery disease. J Eval Clin Pract 21(5):937–942. 10.1111/jep.12409 26137908 10.1111/jep.12409

[23] Oleksiak A, Kępka C, Rucińska K, Marcinkiewicz K, Demkow M, Kruk M (2022) Hemoglobin level as a predictor of major adverse cardiac events during a long-term follow-up in patients with coronary artery disease. Pol Arch Intern Med 21(13212):16377 10.20452/pamw.16377 10.20452/pamw.16377 36468226

[24] Kalra PR, Greenlaw N, Ferrari R, Ford I, Tardif JC, Tendera M et al (2017) Hemoglobin and change in hemoglobin status predict Mortality, cardiovascular Events, and bleeding in stable coronary artery disease. Am J Med 130(6):720–730. 10.1016/j.amjmed.2017.01.002 28109968 10.1016/j.amjmed.2017.01.002

[25] De Luca G, Secco GG, Cassetti E, Verdoia M, Bellomo G, Marino P (2014) Haemoglobin levels do not correlate with the extent of coronary artery disease: results from a large cohort study. Coron Artery Dis 25(6):463–468. 10.1097/mca.0000000000000103 24614627 10.1097/MCA.0000000000000103

[26] Hare GMT, Mazer CD (2021) Anemia: perioperative risk and treatment opportunity. Anesthesiology 135(3):520–530. 10.1097/aln.0000000000003870 34197591 10.1097/ALN.0000000000003870

[27] Kaiafa G, Kanellos I, Savopoulos C, Kakaletsis N, Giannakoulas G, Hatzitolios AI (2015) Is anemia a new cardiovascular risk factor? Int J Cardiol 186:117–124. 10.1016/j.ijcard.2015.03.159 25814357 10.1016/j.ijcard.2015.03.159

[28] Gibbons GH, Dzau VJ (1994) The emerging concept of vascular remodeling. N Engl J Med 330(20):1431–1438. 10.1056/nejm199405193302008 8159199 10.1056/NEJM199405193302008

[29] Rymer JA, Rao SV (2018) Anemia and coronary artery disease: pathophysiology, prognosis, and treatment. Coronary artery disease: pathophysiology, prognosis, and treatment. Coron Artery Dis 29(2):161–167. 10.1097/mca.0000000000000598 29280914 10.1097/MCA.0000000000000598

[30] Chen TK, Knicely DH, Grams ME (2019) Chronic kidney disease diagnosis and management: A review. JAMA 322(13):1294–1304. 10.1001/jama.2019.14745 31573641 10.1001/jama.2019.14745 PMC7015670

[31] Levey AS, James MT (2017) Acute kidney injury. Ann Intern Med 167(9):ITC66–ITC80. 10.7326/aitc201711070 29114754 10.7326/AITC201711070

[32] Pocock S, Bueno H, Licour M, Medina J, Zhang L, Annemans L (2015) Predictors of one-year mortality at hospital discharge after acute coronary syndromes: A new risk score from the EPICOR (long-tErm follow uP of antithrombotic management patterns in acute coronary syndrome patients) study. Eur Heart J Acute Cardiovasc Care 4(6):509–517. 10.1177/2048872614554198 25301783 10.1177/2048872614554198 PMC4657391

[33] Sarnak MJ, Amann K, Bangalore S, Cavalcante JL, Charytan DM, Craig JC (2019) Chronic kidney disease and coronary artery disease: JACC State-of-the-Art review. J Am Coll Cardiol 8(14):1823–1838. 10.1016/j.jacc.2019.08.1017 10.1016/j.jacc.2019.08.1017 31582143

[34] Ataklte F, Song RJ, Upadhyay A, Musa Yola I, Vasan RS, Xanthakis V (2021) Association of mildly reduced kidney function with cardiovascular disease: the Framingham heart study. J Am Heart Assoc 10(16):e020301. 10.1161/jaha.120.020301 34387110 10.1161/JAHA.120.020301 PMC8475034

[35] Ebert T, Neytchev O, Witasp A, Kublickiene K, Stenvinkel P, Shiels PG (2021) Inflammation and oxidative stress in chronic kidney disease and Dialysis patients. Antioxid Redox Signal 10(17):1426–1448. 10.1089/ars.2020.8184 10.1089/ars.2020.8184 34006115

[36] Kaesler N, Babler A, Floege J, Kramann R (2020) Cardiac remodeling in chronic kidney disease. Toxins (Basel) 12(3):161. 10.3390/toxins12030161 32150864 10.3390/toxins12030161 PMC7150902

[37] Suh SH, Kim SW (2023) Dyslipidemia in patients with chronic kidney disease: an updated overview. Diabetes Metab J 47(5):612–629. 10.4093/dmj.2023.0067 37482655 10.4093/dmj.2023.0067 PMC10555535

[38] Cohen E, Margalit I, Shochat T, Goldberg E, Krause I (2019) The relationship between the concentration of plasma homocysteine and chronic kidney disease: a cross sectional study of a large cohort. J Nephrol 32(5):783–789. 10.1007/s40620-019-00618-x 31165981 10.1007/s40620-019-00618-x

[39] Ganguly P, Alam SF (2015) Role of homocysteine in the development of cardiovascular disease. Nutr J 14:6. 10.1186/1475-2891-14-6 25577237 10.1186/1475-2891-14-6 PMC4326479

[40] Abdelmaguid A, Roberts LN, Tugores L, Joslin JR, Hunt BJ, Parmar K (2022) Evaluation of novel coagulation and platelet function assays in patients with chronic kidney disease. J Thromb Haemost 20(4):845–856. 10.1111/jth.15653 35068080 10.1111/jth.15653 PMC9306477

[41] Baaten C, Vondenhoff S, Noels H (2023) Endothelial cell dysfunction and increased cardiovascular risk in patients with chronic kidney disease. Circ Res 132(8):970–992. 10.1161/circresaha.123.321752 37053275 10.1161/CIRCRESAHA.123.321752 PMC10097498

[42] Atkinson MA, Warady BA (2018) Anemia in chronic kidney disease. Pediatr Nephrol 33(2):227–238. 10.1007/s00467-017-3663-y 28412770 10.1007/s00467-017-3663-y

[43] Wu HHL, Chinnadurai R (2022) Erythropoietin-Stimulating agent hyporesponsiveness in patients living with chronic kidney disease. Kidney Dis (Basel) 8(2):103–114. 10.1159/000521162 35527989 10.1159/000521162 PMC9021651

[44] Batchelor EK, Kapitsinou P, Pergola PE, Kovesdy CP, Jalal DI (2020) Iron deficiency in chronic kidney disease: updates on Pathophysiology, Diagnosis, and treatment. J Am Soc Nephrol 31(3):456–468. 10.1681/asn.2019020213 32041774 10.1681/ASN.2019020213 PMC7062209

[45] Ow CPC, Ngo JP, Ullah MM, Hilliard LM, Evans RG (2018) Renal hypoxia in kidney disease: cause or consequence? Acta Physiol (Oxf) 222(4):e12999. 10.1111/apha.12999 29159875 10.1111/apha.12999

[46] Wang B, Li ZL, Zhang YL, Wen Y, Gao YM, Liu BC (2022) Hypoxia and chronic kidney disease. EBioMedicine 77:103942. 10.1016/j.ebiom.2022.103942 35290825 10.1016/j.ebiom.2022.103942 PMC8921539

[47] Io H, Aizawa M, Funabiki K, Horikoshi S, Tomino Y (2015) Impact of anaemia treatment for left ventricular remodelling prior to initiation of Dialysis in chronic kidney disease patients: efficacy and stability of long acting erythropoietin stimulating agents. Nephrol (Carlton) 20(Suppl 4):7–13. 10.1111/nep.12640 10.1111/nep.12640 26439537

[48] Chang JM, Chen SC, Huang JC, Su HM, Chen HC (2014) Anemia and left ventricular hypertrophy with renal function decline and cardiovascular events in chronic kidney disease. Am J Med Sci 347(3):183–189. 10.1097/MAJ.0b013e31827981be 23426086 10.1097/MAJ.0b013e31827981be

[49] Alamin AA, Yahia AIO, Hussien HM (2025) Red blood cells in thrombosis: active participants in clot formation and stability: a systematic review. Semin thromb Hemost 10.1055/a-2552-9525 10.1055/a-2552-9525 40154506

[50] Tapio J, Vähänikkilä H, Kesäniemi YA, Ukkola O, Koivunen P (2021) Higher hemoglobin levels are an independent risk factor for adverse metabolism and higher mortality in a 20-year follow-up. Sci Rep 11(1):19936. 10.1038/s41598-021-99217-9 34620927 10.1038/s41598-021-99217-9 PMC8497471

[51] Chen ZQ, Luo L, Chen XX, Zhang XY, Yin SQ, Xiao GH et al (2024) Dietary nutrient intake and nutritional status in maintenance Hemodialysis patients: a multicenter cross-sectional survey. Ren Fail 46(2):2363589. 10.1080/0886022X.2024.2363589 38874093 10.1080/0886022X.2024.2363589 PMC11182067

[52] Kashani K, Rosner MH, Ostermann M (2020) Creatinine: from physiology to clinical application. Eur J Intern Med 116:168–169. 10.1016/j.ejim.2019.10.025 10.1016/j.ejim.2019.10.025 31708357

