# Supplementary material for: Association between hemoglobin-to-creatinine ratio and all-cause mortality in patients with coronary artery disease
Source: Egypt Heart J. 2025 Dec 5;77:109. doi: 10.1186/s43044-025-00707-2 (PMC12680590; doi:10.1186/s43044-025-00707-2)

**Supplemental Table 1. VIF of covariates**

| **Covariates** | **VIF** |
| --- | --- |
| **HCR** | 2.9 |
| **Sex** | 1.5 |
| **Age** | 1.3 |
| **Race** | 1.1 |
| **Education level** | 1.2 |
| **Ratio of family income to poverty** | 1.2 |
| **BMI** | 1.2 |
| **Alcohol use** | 1.2 |
| **Smoking** | 1.1 |
| **ALT** | 1.9 |
| **AST** | 1.9 |
| **Uric Acid** | 1.3 |
| **Hemoglobin** | 1.7 |
| **Creatinine** | 1.8 |
| **Hypertension** | 1.1 |
| **Hypercholesterolemia** | 1.1 |
| **Diabetes** | 1.1 |
| **Heart Failure** | 1.1 |
| **Stroke** | 1.0 |

**Supplemental Fig 1. Smooth curve fitting**

**
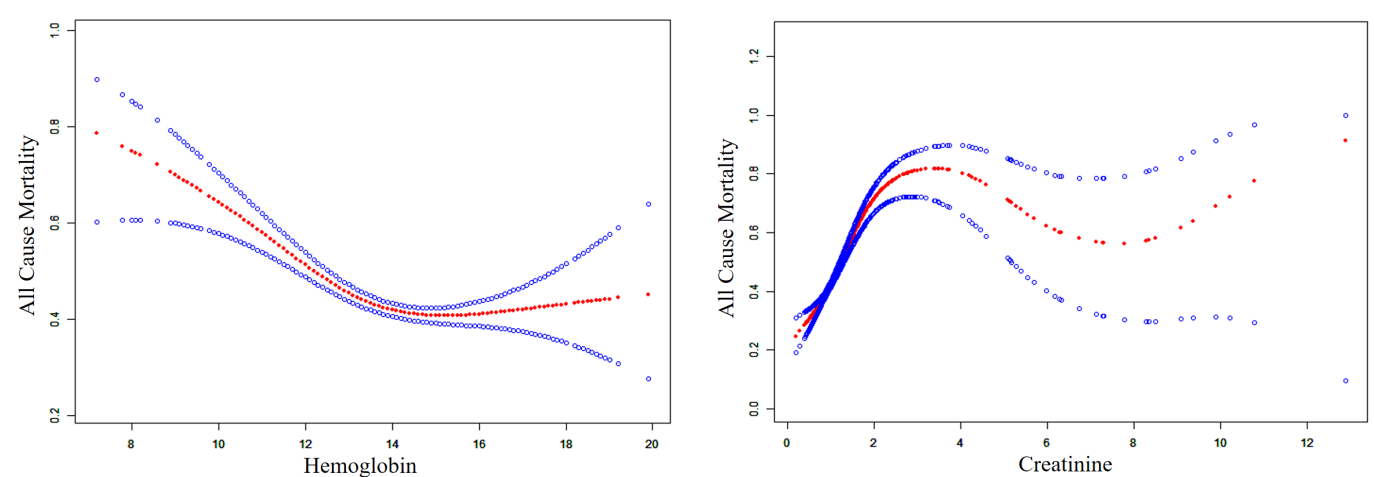
**

**Supplemental Table** 2. Threshold saturation effect analysis

|  | **Hemoglobin** | **Creatinine** |
| --- | --- | --- |
|  | **HR（95%CI）P** | **HR（95%CI）P** |
| **K** | 13.2 | 3 |
| **＜K** | 0.74 (0.68, 0.81) <0.0001 | 1.67 (1.28, 2.17) 0.0001 |
| **≧k** | 0.99 (0.94, 1.06) 0.8431 | 1.15 (1.01, 1.30) 0.0341 |
| **P** for Logarithmic likelihood ratio test | <0.001 | 0.034 |

Adjusted for sex, age, race, education level, ratio of family income-to-poverty, BMI, smoking, alcohol use, ALT, uric acid, iron, albumin, hemoglobin, creatinine, hypertension, hypercholesterolemia, diabetes, heart failure, and stroke (except the independent variable itself).

**Supplemental Fig 2. Receiver operating characteristic curves**


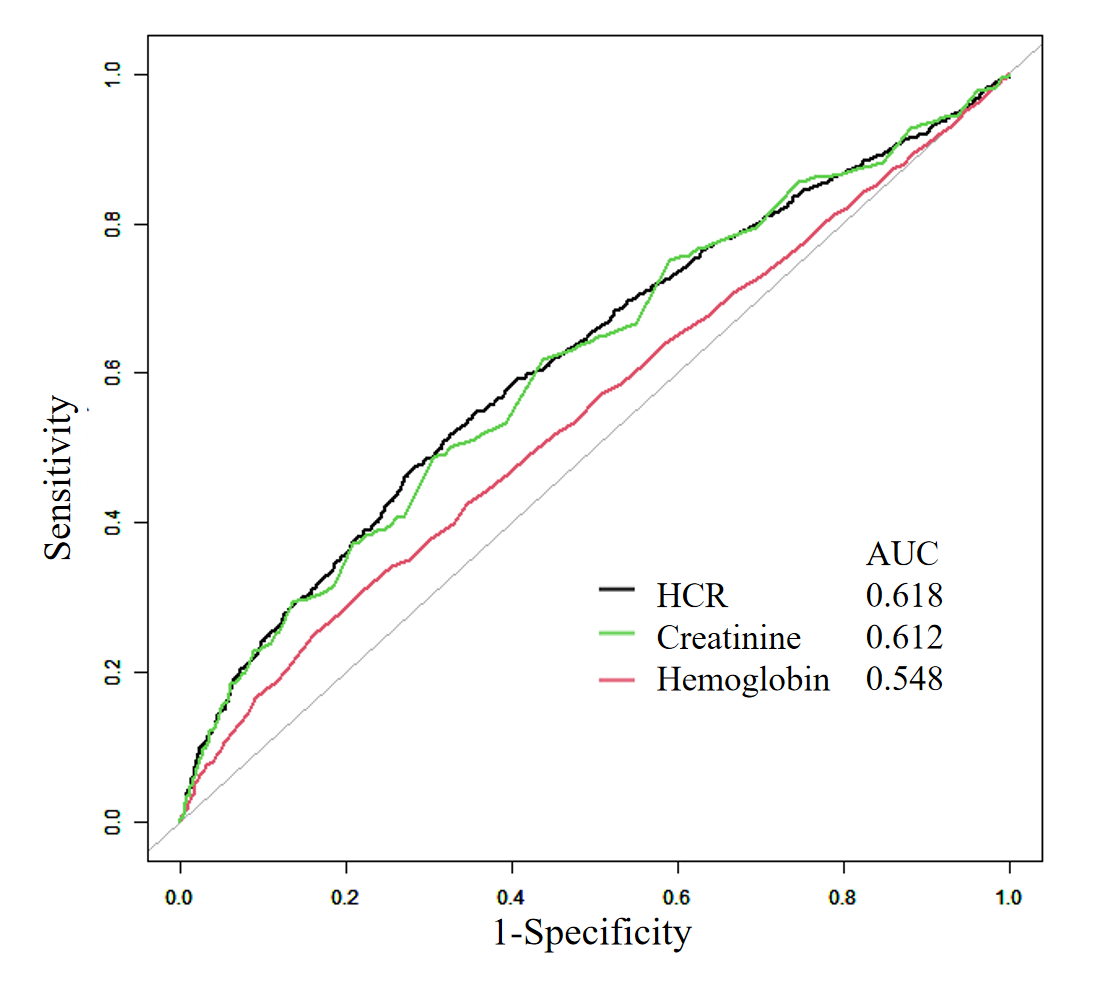

Supplement: Supplementary file 2 — Supplementary Material 2. [file 43044_2025_707_MOESM2_ESM.doc]
